# First documented case of Smith-Lemli-Opitz syndrome in Syria: clinical presentation, diagnosis, and experimental management with simvastatin

**DOI:** 10.1093/omcr/omae129

**Published:** 2024-11-20

**Authors:** Alwa Hussien Aladia, Samar Hamdan, Ahmad Alkheder

**Affiliations:** Department of Pediatric, Children University Hospital, Faculty of Medicine, Damascus University, Damascus, Syria; Department of Pediatric, Syrian Mouasat Association Hospital, Damascus, Syria; Faculty of Medicine, Al- Baath University, Homs, Syria; Department of Pediatric, Children University Hospital, Faculty of Medicine, Damascus University, Damascus, Syria; Faculty of Medicine, Tishreen University, Lattakia, Syria; Department of Otorhinolaryngology, Al Mouwasat University Hospital, Faculty of Medicine, Damascus University, Damascus, Syria; Faculty of Medicine, Syrian Private University, Damascus, Syria

**Keywords:** Smith-Lemli-Opitz syndrome, genetic disorder, first case, 7-dehydrocholesterol, simvastatin, SLOS

## Abstract

Smith-Lemli-Opitz syndrome (SLOS) is a rare genetic disorder that affects cholesterol synthesis and causes various physical and mental abnormalities. The case is a 25-day-old male infant who presented with multiple congenital anomalies, such as microcephaly, facial dysmorphism, syndactyly, hypospadias, and other organ malformations. He also had severe vomiting, feeding difficulty, irritability, dehydration, and hyponatremia. Laboratory tests showed low serum cholesterol, in addition to genetic tests, confirming the diagnosis of SLOS. The infant was treated with simvastatin, which improved his irritability and was well tolerated. The paper discusses the clinical features, diagnosis, and management of SLOS, and highlights the importance of early recognition and intervention for this rare case. It is also considered the first documented case in Syria.

## Introduction

Smith-Lemli-Opitz syndrome (SLOS) is an inherited metabolic disorder affecting cholesterol synthesis. It stems from mutations in the gene encoding 7-dehydrocholesterol (DHCR7) reductase, leading to disrupted cholesterol biosynthesis, thus presenting as a deficiency in cholesterol production. Consequently, affected individuals exhibit reduced cholesterol levels alongside elevated DHCR7 levels, precipitating various clinical manifestations. The syndrome’s prevalence ranges from approximately 1 in 20 000 to 1 in 70 000, with a higher incidence observed among individuals of European ancestry. Manifestations include a spectrum of developmental and physical anomalies, encompassing intellectual disability, distinct facial characteristics, and structural abnormalities in organs [1, 2].

In our report, we present a rare case of SLOS, which is considered the first documented case in Syria.

## Case presentation

A 25 days old male infant was the first born child of a 26 year old mother and 32 year old father. Family history was unremarkable and there was no parental consanguinity.

During pregnancy, the pregnancy was well monitored. The mother denied any history of drug abuse involving tobacco and ethanol. Furthermore, there was no reported exposure to radiation or chemical substances. It is noted that the mother received antibiotic therapy during the third and fourth months of gestation due to recurrent urinary tract infections. Congenital abnormalities have been detected in the fetus at 33 weeks of gestation, including:Atrial septal defect$\,{{{\cdot}}\!\! {\hbox{`}} } $ shortened lower limbs, narrow forehead, small jaw, and signs of restricted fetal growth in the womb.

During Postnatal course, the birth length was 46 cm, weight 2000 g and head circumference 33 cm. The baby was admitted to hospital with severe vomiting, feeding difficult and irritability, with multiple congenital anomalies were noted by the family.

**Figure 1:**
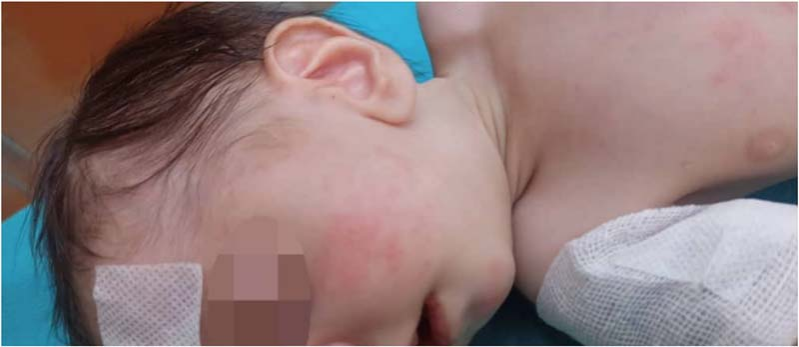
Lateral view of the head, showing the low position of the ear.

**Figure 2:**
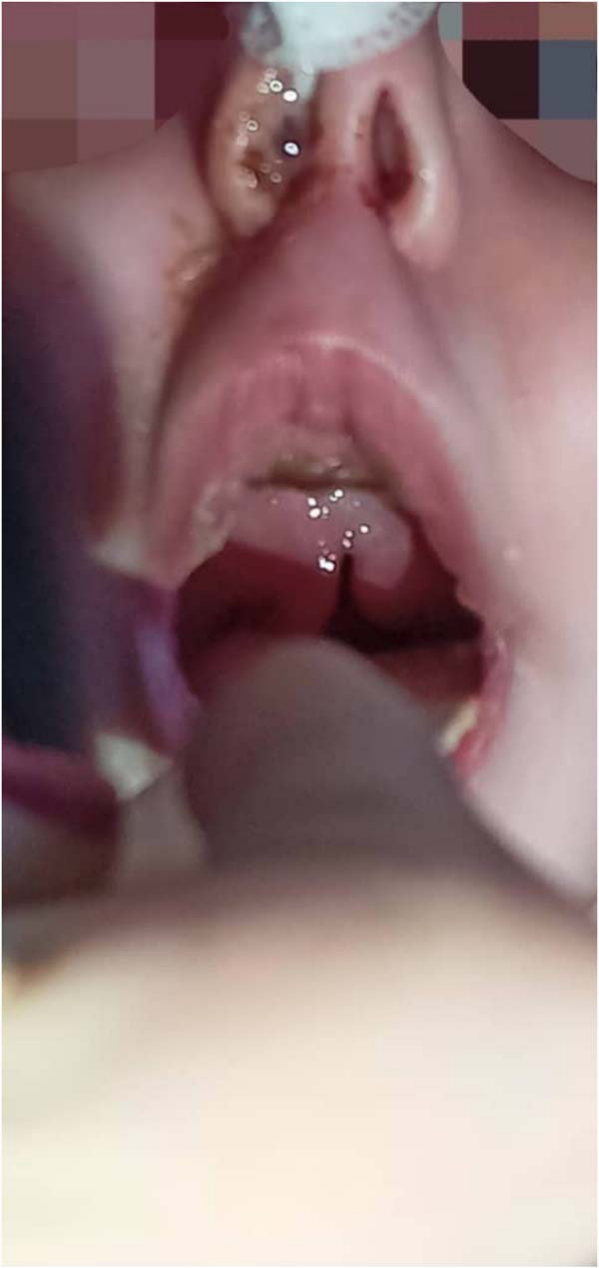
Anterior view of the head with the mouth open, showing staphyloschisis.

**Figure 3:**
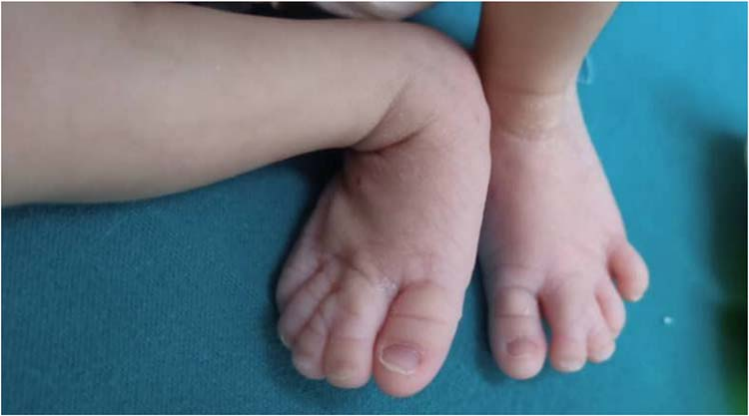
A view of the lower extremities, showing the hadsyndactyly of the second and third toes.

**Figure 4:**
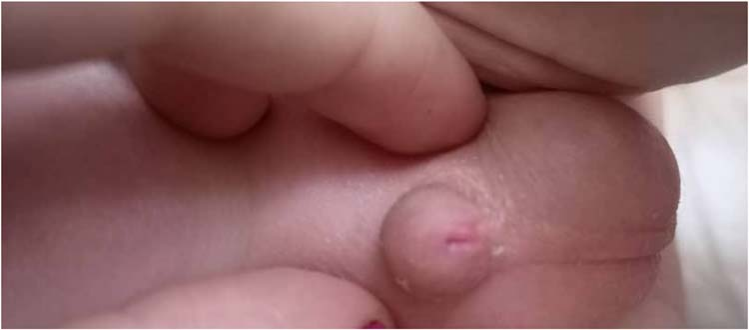
A view of the genital area, showing small penis hypospadiasis.

Physical examination: the infant had microcephaly, broad nasal tip, maxillary hypoplasia gingival enlargement, low set ears, (Fig. 1) ptosis and staphyloschisis (Fig. 2), the limbs hadsyndactyly of second and third toes (Fig. 3). Cardiovascular system examination showed short systolic murmure at lower left sternal borderin. Genital examination showed small penis hypospadiasis with undescended testis (Fig. 4). The baby had dry mucous membranes and tachycardia.

Laboratory examination and diagnostic tests, the laboratory investigations showed hyponatremia and elevated level of blood urea. Routine peripheral blood cell counts were normal. Serum cholesterol level showed low value of 43 mg/dl (normal value in neonates 85–165 mg/dl [3]).

### Karyotyping was 46XY

Our patient is homozygous for a pathogenic variant, [NM-001360.2:c121OC,T;p.(Arg404CYS)] in the DHCR7 gene. Pathogenic variants in the DHCR7 gene are associated with Smith-Lemli-Optiz syndrom with an autosomal recessive mode of inheritanc.

**Figure 5:**
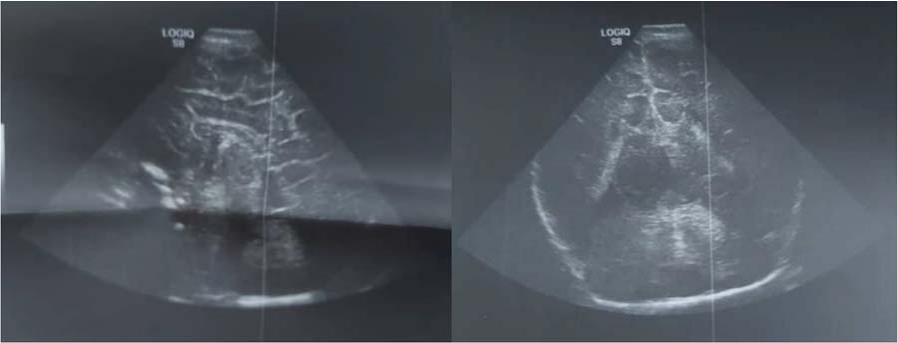
Ultrasound of head shows agenesis of corpus callosum.

Radiological studies, the infant was evaluated with echocardiography that showed atrial septal defect (ASD) with pulmonary hypertension. The head ultrasound showed a agenesis of corpus callosum (Fig. 5), abdominal ultrasonography showed Horseshoe kidney. Upper gastrointestinal contrast study showed sevrs gastro oesophageal reflux. A diagnosis of Smith-Lemli-Opitz-syndrome was made based on clinical ، biochemical profile and molecular genetic testing.

Then, we managed dehydration and hyponatremia. An orogastric tube was inserted and Tips were provided to parents for managing gastroesophageal reflux, including elevating the head of the bed, feeding in an upright position, and avoiding tight clothing. After our patient experienced severe irritability, he was prescribed simvastatin at a dose of 0.5 mg/kg/day for 6 weeks, followed by an increased dose of 1 mg/kg/day for 12 months. During the follow-up period, an improvement in the baby’s irritability was observed with good tolerance and without any side effects.

## Discussion

Smith-Lemli-Opitz syndrome (SLOS) is an autosomal recessive disorder characterized by a deficiency in the enzyme critical for the final step of cholesterol synthesis [1].

This syndrome presents with a spectrum of symptoms, encompassing intellectual disability, developmental delays, and distinct facial features such as microcephaly, widened eyes, and a broad nasal bridge. Additionally, individuals may exhibit cardiac abnormalities, growth and feeding difficulties, behavioral issues, autism spectrum disorder, and hypotonia [1].

The severity of SLOS varies widely among affected individuals, ranging from mild to severe complications. Diagnosis typically involves clinical evaluation, biochemical assessment of cholesterol and 7-dehydrocholesterol levels, and genetic analysis to detect mutations in the DHCR7 gene [3,4].

As of now, there is no established long-term treatment for SLOS [5]. However, management strategies may include:

1. Cholesterol supplementation: Since individuals with this syndrome have impaired cholesterol synthesis, supplementation with cholesterol precursors such as dietary cholesterol or cholesterol-containing medications may be recommended.
2. Management of developmental delays: Timely access to early intervention programs, along with tailored regimes of physical therapy, occupational therapy, and speech therapy, could facilitate the amelioration of developmental delays, thereby enhancing holistic functionality.
3. Management of feeding difficulties: Some individuals with Lemli-Smith Opitz syndrome may have feeding difficulties due to oral-motor dysfunction or swallowing problems. Working with a speech therapist or a nutritionist can help address these issues.
4. Monitoring and management of congenital heart defects: regular monitoring and treatment of any heart defects present in individuals with Lemli-Smith Opitz syndrome are essential to prevent complications.
5. Genetic counseling: engaging in genetic counseling offers families insights into the hereditary transmission mechanisms associated with the syndrome, furnishing valuable information pertaining to the likelihood of recurrence in subsequent offspring.

Recent findings have indicated that simvastatin treatment led to enhancements in the serum dehydrocholesterol-to-total sterol ratio and resulted in a significant decrease in irritability symptoms among individuals diagnosed with mild spectrum Smith-Lemli-Opitz syndrome [6].

It’s important to note that treatment may vary depending on the specific symptoms and complications experienced by each individual with Lemli-Smith Opitz syndrome. Therefore, it is essential for patients to work closely with a multidisciplinary healthcare team that includes specialists in genetics, cardiology, neurology, and other relevant fields.

## Conclusion

In cases featuring syndactyly of the 2nd and 3rd toes and facial dysmorphism, careful consideration is warranted. Future endeavors in the realm of Smith-Lemli-Opitz syndrome (SLOS) encompass the development of novel diagnostic modalities characterized by confirmatory capability, cost-effectiveness, rapidity, and heightened sensitivity and specificity, while also being user-friendly. The therapeutic approach to this syndrome primarily revolves around the management of associated symptoms and complications. While simvastatin has exhibited promise in enhancing outcomes among pediatric SLOS patients, further investigation is imperative to comprehensively elucidate its long-term ramifications and determine optimal dosing regimens within this demographic.

## Supplementary Material

Cover_Letter_omae129
