# Supplementary material for: First documented case of Smith-Lemli-Opitz syndrome in Syria: clinical presentation, diagnosis, and experimental management with simvastatin
Source: Oxf Med Case Reports. 2024 Nov 20;2024(11):omae129. doi: 10.1093/omcr/omae129 (PMC11576548; doi:10.1093/omcr/omae129)
Supplement: Cover_Letter_omae129 [file cover_letter_omae129.docx]

# **Date: 5/3/2024 Journal name: Oxford Medical Case Reports**

**Oxford Medical Case Reports – Decision on Manuscript ID OMCR-2024-131**

# I am enclosing here with a manuscript entitled **“First Documented Case of Smith-Lemli-Opitz Syndrome in Syria: Clinical Presentation, Diagnosis, and Successful Management with Simvastatin”** for publication in **Oxford Medical Case Reports** for possible evaluation. The Corresponding author of this manuscript is **Ahmad Alkhede**r and the authors .

| Sl no | Author full name | Email ID | address |
| --- | --- | --- | --- |
| 1 | Alwa Hussien Aladia | alowaadia1993@gmail.com | Damascus, Syria |
| 2 | Samar Hamdan | Samar.hamdan799@gmail.com | Damascus, Syria |
| 3 | Ahmad Alkheder | alkhederahmed@gmail.com | Damascus, Syria |

1. **UNDERTAKING**

With the submission of this manuscript I would like to undertake that:

- All authors of this Article (paper) have directly participated in the planning, execution, or analysis of this study;
- All authors of this paper have read and approved the final version submitted;
- The contents of this manuscript have not been copyrighted or published previously;
- The contents of this manuscript are not now under consideration for publication elsewhere;
- The contents of this manuscript will not be copyrighted, submitted, or published elsewhere, while acceptance by the Journal is under consideration;
- There are no directly related manuscripts or abstracts, published or unpublished, by any authors of this paper;

**2. GRANTS OR FUNDING INFORMATION**

No funding was received for this research.

Date: 5/3/2024

_________________________________________________
